# Malaria Outbreak Investigation in Chipinge, Zimbabwe: A Case-control Study

**Published:** 2017

**Authors:** Tinashe KUREYA, Augustine NDAIMANI, Maxwell MHLANGA

**Affiliations:** 1. Provincial Medical Directorate, Ministry of Health and Child Care, Mutare, Zimbabwe; 2. Dept. of Nursing Science, College of Health Sciences, University of Zimbabwe, Harare, Zimbabwe

**Keywords:** Case-control, Malaria, Manicaland, Outbreak investigation

## Abstract

**Background:**

Malaria outbreaks are common in Zimbabwe. They are common in Manicaland, which has the greatest burden of malaria in the country.

**Methods:**

A matched case control study was conducted to investigate the malaria outbreak in ward 13 and 14 of Chipinge district in Manicaland Province in Zimbabwe, week 30 to week 40 of year 2015. A sample size of 92 (46 cases and 46 controls) was used. Guided interviews were conducted with the aid of a structured questionnaire and a checklist. The investigation assessed factors associated with contracting malaria and the community knowledge levels on malaria.

**Results:**

Participants who stayed in houses with open eaves had 2.4 odds (95% CI=1.0; 5.6) of contracting malaria compared to those who lived in houses without open eaves. Staying within a radius of 3 km from the river or swamp also predisposed people to contracting malaria (OR =2.7, 95%CI=1.2; 6.3). People who had no insecticide treated mosquito nets hanged in their bed rooms had odds of 2.2 (95%CI=1.2; 6.4) of contracting malaria compared to those that hanged insecticide-treated mosquito nets in their bedrooms. Consequently, among people exposed to outdoor activities in the evening and at night, those that had insecticide-treated mosquito nets hanged in their rooms were more protected from malaria than those that did not.

**Conclusion:**

There is high need to intensify all pillars in the malaria prevention and control programs and maintenance of a strong surveillance system to prevent future occurrences of outbreaks.

## Introduction

In the world currently, an estimated 3.2 billion people are at the risk of being infected with malaria and developing the disease. According to latest reports 198 million cases of malaria occurred globally in 2013 and these led to 580 000 deaths. Malaria burden is greatest in the WHO African region where an estimated 90% of all malaria deaths occur and children aged under 5 years accounts for 78% of deaths (1).

Zimbabwe has seen a significant decline in malaria transmission and burden. Today, malaria is the 5^th^ leading cause of morbidity (compared to 2^nd^ leading cause death in 2013).” In year 2013, incidence was reported at 29 per 1000 down from 58 per 1000 in 2009, 351 deaths were recorded (down from 375 deaths in 2009) (1). Zimbabwe has 10 provinces and 65 rural districts, 47 of them are considered malarious while 30 have a high malaria burden, Chipinge district included. In the year, 2013 of all the malaria cases recorded in Zimbabwe 35% of malaria deaths were from Manicaland province (1).

A number of interventions have been launched for malaria prevention treatment and care at provincial and at district level. As a result, a lot of significant changes have been documented in line with the progress. Chipinge district has recorded an increase in IRS (In door Residual Spraying) coverage from 83% in 2013 to 91% in 2014. However, IPTp (Intermittent Preventive Treatment in pregnancy) coverage for pregnant women dropped from 86.8% to 70.1%. Malaria surveillance, completeness of reporting (T5) increased from 97% to 99% from year 2013 to year 2014 (1).

According to the Manicaland Province Malaria report of 2015, the period January to June 2015 recorded 63525 suspected malaria cases and 60560 tested cases (2). The percentage test rate was 95.3% and the total number of confirmed cases was 28176. The target of malaria diagnosis was at least 95%, parasitological testing of suspected cases in the community and January to June 2015 recorded coverage of 98.5%^2^. Consequently, the target for IPTp coverage in the province is that above 80% of pregnant women should receive at least 2 doses of IPTp, the first 2 quarters of 2015 recorded coverage of 79.7% (2).

Completeness of reporting, the target for completeness based on T5 forms should be at least 95%, but 2013 recorded 97%, year 2014 recorded 99% and the first 2 quarters of 2015 recorded 99%^2^. Completeness of Village Health Worker reports has a target of at least 95%, year 2013 recorded a completeness of 2.9%, year 2014 recorded 15.6% and the first 2 quarters of 2015 had a completeness of 59.6% (2).

This study sought to investigate the causes of the outbreak in Kopera Clinic Catchment area. The focus was on factors associated with contracting Malaria, describing the outbreak by person place and time and making use of the findings to institute control and preventive measure.

## Methods

A matched case control design was used. Cases were people who presented at Kopera Clinic in Chipinge east (Manicaland Province) confirmed as having malaria through a positive parasitological test; either a Rapid Diagnostic Test (RDT) or positive Malaria Parasite Slide (MPS). Matching was based on demographic variables. Controls were people who presented to Kopera Clinic in the period between week 30 and week 40 in 2015 but got a negative malaria result based on a parasitological test. Cases and controls were selected from patients who presented at the clinic between week 30 and 40 in 2015. A sample of 46 cases and 46 controls was selected using T12 registers. Sample size was calculated using Epi info 7, two sided confidence level of 95%, Power 80% and the assumption of the percentage of controls exposed to pole and dagga houses is 16%. The methods used in this outbreak investigation are based on the updated guidelines of investigating an outbreak published by the Centers for Disease Control and Prevention (CDC). The guidelines describe the critical stages an outbreak investigation should follow (2).

Permission to conduct the study was granted by the Provincial Medical Director (PMD) through the District Medical Officer (DMO). The study was cleared by Africa University research and Ethic Committee (AUREC). Informed consent was also sought from participants before collecting data.

Pretesting of instruments was done on 8 respondents from 2 villages, respondents who participated in the pretesting were not interviewed in the main research project; the process checked for reliability and validity of instruments. Guided Interviews were conducted with the aid of structured questionnaires to 46 cases and 46 controls from ward 13 and ward 14. A checklist was also used to measure availability of malaria case management resources at Kopera Clinic. Data was analyzed using Epi-Info 7.

## Results

There were 39 (42.4%) males and 53(57.6%) females in the study. Most of the participants, 42(45.7%) were aged between 18 to 59 year. Sixteen (17.4%) had no formal education. Most of those without formal education were in the under-five and 65 or above age group. Forty-two participants (45.6%) were married. The rest were single, widowed or divorced. There was 1 (1.1%) professional.

**Fig. 1:**
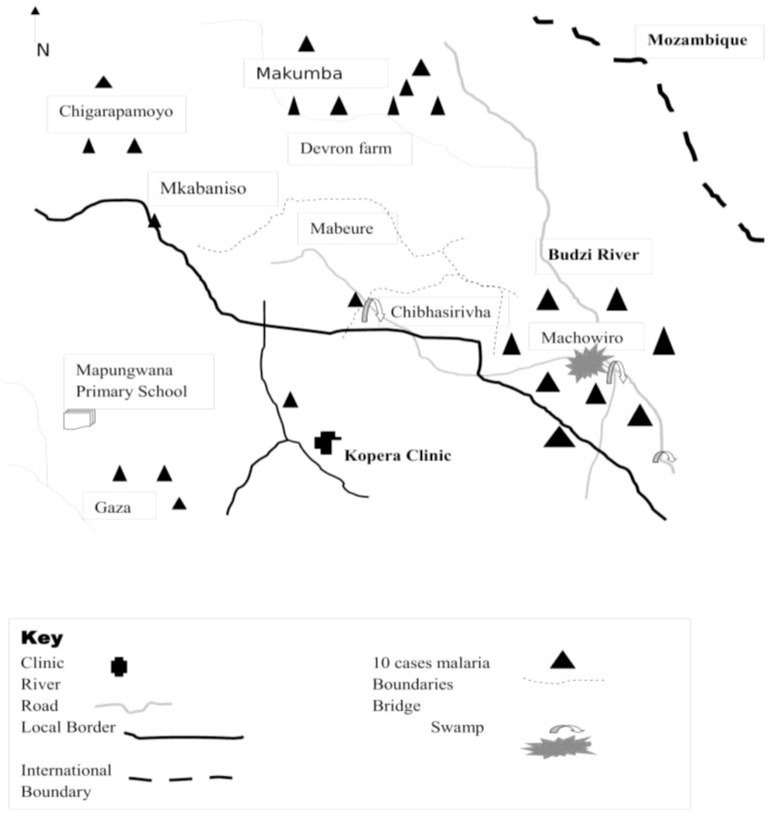
Spot-map (Distribution of Malaria Cases)

Twenty-three (25%) were cases affiliated to Apostolic churches, 5 (5.4%) to African Traditional religion (ATR) while 15 (16.3%) were among controls affiliated to Protestant churches and 17 (18.6%) to Apostolic churches. Hence, most cases were female, aged between 5–18 years, people with only primary education, married, and farmworkers and of Apostolic religion. Demographic characteristics of the sample are summarized in Table 1 below. Ward 13 and 14 are part of the catchment area of Kopera Clinic. Most cases were recorded from Machowiro villages followed by Makumba, Chigarapamoyo, Mabeure and others. In terms of Malaria case management; there are 3 Village Health Workers in Machowiro villages and Makumba village who recorded the most cases in week 31 to week 40. Budzi River and Machowiro swamp are common features in the 2 wards. Since this was late in the dry season most water related activities such as bathing, fishing, laundry, watering crops and animals were concentrated along the river and around the swamp. The spot map of the 2 wards is shown in Fig. 1.

**Table 1:** Sample Demographics (N=92)

***Variable***		***Case (%)***	***Controls (%)***
**Sex**	Male	17 (37)	22 (48)
	Female	29 (63)	24 (52)
**Age (completed years)**	Under 5	8 (17)	5 (11)
	5–18	15 (33)	13 (28)
	18–59	17 (37)	25 (54)
	65^+^	6 (13)	3 (07)
**Highest level of Education**	None	9 (20)	7 (15)
	Primary	25 (54)	24 (52)
	Secondary	8 (17)	9 (19)
	Tertiary	4 (09)	6 (13)
**Marital status**	Single	17 (37)	13 (28)
	Divorced	4 (09)	2 (04)
	Married	19 (19)	23 (05)
	Widowed	6 (13)	8 (17)
**Occupation**	Farmer	9 (20)	17 (37)
	Farm workers	11 (25)	5 (11)
	Professional	0 (00)	1 (02)
	Casual Laborer	9 (20)	14 (30)
	Not working	17 (37)	9 (20)
**Religion**	ATR	5 (5.4)	3 (3.3)
	Pentecostal	6 (6.5)	11 (12)
	Protestant	9 (9.8)	15 (16.3)
	Apostolic	23 (25)	17 (18.6)
	Other	3 (3.3)	0

### Description of the Outbreak

**Fig. 2:**
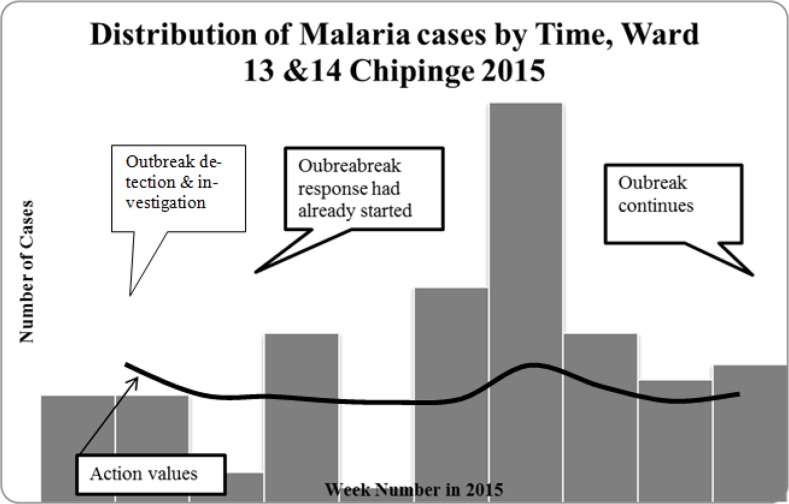
Epidemic Curve showing malaria cases week 31 to week 40 year 2015: based on Chipinge district Malaria Threshold values for 2015

The outbreak started in week 10 of this 2015 and it peaked in week 15 then dropped off in week 29. In week 31, it started again but at a lower intensity than in week 10 to week 29. In this current study period, the highest peak was in week 37. The outbreak dropped down in week 40 although it continued thereafter. Across this period, the health team had started some awareness campaigns and the Village Health Workers were equipped with RDT Kits, registers and malaria treatment medical drugs. The epidemic curve shows that the type of outbreak under study is a continuous source outbreak since the cases arise over an extended period but still originate from a common or single source. The Epi-curve is longer and flatter indicating the longer duration of the source and the variations in incubation periods between people, Fig 2.

Statistically significant correlates of malaria contraction included: living in a house with open eaves (OR = 0.7; 95% CI = 0.3; 1.8), staying within 3 kilometers of the river or swamp (OR=2.7; 95% CI = 1.2; 6.3), not having insecticide treated nets (ITNs) at home (OR = 2.2; 95% CI=1.0; 5.1), ITN not hanged in the room (OR=2.7; 95% CI =1.2; 6.4) and having visited Mozambique in the past 6 week (OR=9.5; 95% CI=1.1; 79.2). The later had a wide confidence interval, Table 2.

**Table 2:** Factors associated with contracting Malaria

***Risk Factors***	***Cases n (%)***	***Controls n (%)***	***Odds Ratio (95% CI)***
**Living in Houses of Pole and Dagga**	30 (65)	33 (72)	0.7(0.3; 1.8)
**House made of Dagga and Bricks**	16 (35)	13 (28)	
**Living in House with open eaves**	29 (63)	19 (41)	2.4 (1.0; 5.6)
**House without open eaves**	17 (37)	27 (59)	
**Outdoor Activities in the evening**	33 (72)	28 (64)	1.5 (0.6; 3.5)
**No outdoor activities, evening**	13 (28)	16 (36)	
**Wearing short clothes**	21 (53)	26 (59)	0.8 (0.3; 1.8)
**Wearing long clothes**	19 (47)	18 (41)	
**Live 3Km within River or Swamp**	31 (67)	20 (43)	2.7 (1.2; 6.3)
**Live away from River or Swamp**	15 (33)	26 (57)	
**Not Having ITNs at home**	27 (59)	18 (39)	2.2 (1.0; 5.1)
**Having ITNs at Home**	19 (41)	28 (61)	
**ITN not hanged in the room**	32 (70)	21 (46)	2.7 (1.2; 6.4)
**ITN hanged in the room**	14 (30)	25 (54)	
**Having Evening Meals outdoors**	19 (41)	24 (52)	0.6 (0.3; 1.5)
**Evening meals indoors**	27 (59)	22 (48)	
**Having visited Mozambique**	8 (17)	1 (2)	9.5 (1.1; 79.2)
**Not having visited Mozambique**	38 (83)	45 (98)	

There was no statistically significant difference between the stratum specific ORs hence there was no effect modification. Moreover, the crude OR fell within stratum specific ORs hence there was no confounding. People who had outdoor activities in the evening those who hanged ITN nets in their rooms were more protected from contracting malaria than those that did not. The stratified analysis of having outdoor activities with ITNs hung indoors is summarized in Table 3. There was no statistically significant correlation between knowledge and having malaria. Results of the regression are shown in Table 4.

**Table 3:** Stratified analysis of having activities in the evening and having an ITN hanged in the room.

***Variable***			***Outdoor activities in the evening***		***OR***	***95% CL***
				Cases	Controls		
**Outdoor activities**	ITN hanged	Yes	12	17	0.5	(0.2; 1.3)
		No	21	14		
			33	21		
**No outdoor activities**	ITN not hanged	Yes	6	17	0.4	(0.1 1.6)
		No	7	8		
			13	25		
**Crude**		Yes	18	34	0.4	(0.2; 0.9)
		No	28	22		

**Table 4:** Knowledge of Participants and contracting Malaria

	***Risk Factors n (%)***	***Cases n (%)***	***Controls n (%)***	***Odds Ratio (95% CI)***
	**No HE on Malaria in past 6mnths**	8 (18)	14 (30)	0.1 (0.2; 1.4)
	**HE in Malaria past 6 months**	36 (82)	32 (70)	
	**Recall < 3 Signs & Symptoms**	17 (36)	10 (22)	2.1 (0.8; 5.3)
	**Recalled ≥ 3 Signs and symptoms**	29 (63)	36 (78)	
	**Malaria, mangoes & other sources**	7 (15)	3 (7)	0.5 (0.1; 2.0)
	**Malaria, mosquito bites**	41(85)	38 (93)	

### Health Facility preparedness for the outbreak

Most medical essentials for malaria management were available but there were some consumables that were below the minimum stock such as Clindamycin. However, Diazepam, Doxycycline, 5% dextrose and Urostix® were not available in stock. Table 5 below gives a summary of the expected quantities of each of the medical equipment and the required minimum stock.

**Table 5:** Medical facilities available at Kopera Clinic from week 30 to week 40 in 2015

***Item***	***Available***	***Available in stock (1 unit 1= tin =300tablets)***	***Minimum Stock***	***Comments***
**RDT Kits**	Yes	281	20	05/17
**Blood slides**	Yes	Yes	Exceed	Available
**Thermometer**	Yes	6	6	Available
**Sphygmomanometer**	Yes	2		Available
**Urostix**	No			Available
**Clindamycin**	No	0	1	NA
**Quinine (Oral)**	Yes	3	1	07/17
**Quinine (Injectable)**	Yes	40	2	04/17
**Fansidar®**	Yes	7	5	04/18
**Diazepam**	No	0	2	NA
**Doxycycline**	No	0	3	NA
**5% Dextrose**	No	0	1	NA
**50% Dextrose**	Yes	1	1	5/19
**Syringes**	Yes	500	100	03/19
**Needles**	Yes	500	100	05/19
**Intravenous Cannulas**	Yes		8	12/19
**Fluid giving sets**	Yes	15	4	02/17

Lastly, the health team at Kopera Clinic had done community awareness meetings on Malaria, malaria case detection was done using RDT and free treatment was being offered. Larviciding had already been done but Indoor Residual Spraying (IRS) and identification of breeding sites for mosquitoes had not been done in the 2 wards. Outbreak response measures are summarized in Table 6 below.

**Table 6:** Outbreak response measures taken

***Outbreak Response Measures***	***Whether Done or Not (Yes/No)***
**Community Meetings**	Yes
**Testing for Malaria using RDT kits**	Yes
**Free treatment for Malaria**	Yes
**Identification of Mosquito breeding sites**	No
**Indoor residual spraying (IRS)**	No
**Larviciding**	Yes

## Discussion

### Demographics

The findings show that the cases were predominantly females between 18 to 59 years of age groups. Such a trend is similar to the findings (3,4). In these two studies, the cases had more females than males. This may be attributed to the fact that the Zimbabwean population has more females than males. Alternatively, it can also be because adult women do more activities that expose them to mosquito breeding sites, such as fetching water from various water sources.

A study on malaria prevalence and socio-demographic factors in Tanzania found out that children and female household members were more susceptible to attack by malaria (5). In terms of religion, the apostolic sect was the most dominant religious denomination and this was specifically the white garment churches (3). In other studies of such groups have been associated with low health seeking behavior and they have been known for the practice of evening or overnight open-air church gatherings (3).

### Geographical Context in relation to the Spot-map

Most malaria cases were from ward 14 followed by ward 13. In ward 14, most cases came from the 3 villages (namely Machowiro 1, 2 and 3). In ward 13, most cases came from Makumba village. Common geographical features are Budzi River, the perennial swamp in Machowiro village, small-scale tea estates by out-growers and the Zimbabwe-Mozambique border zone. Makumbe village is dominated by small-scale communal farmers newly resettled hence they are still in the process of constructing decent accommodation structures. Thus, most houses are built on pole and dagga and most structures lack windows but they have open eaves for ventilation. Ward 13 and 14 are mostly A1, A2 farms and communal farming areas. Machowiro and Makumba villages are characterized by irregular steep terrain that has a history of being a barrier to 100% IRS coverage in 2012.

### Factors associated with contracting Malaria

Living in a house made up of pole and dagga was found to be protective from contracting malaria compared to living in a house made up of brick and dagga. This is contradictory to the findings of the malaria outbreak investigation in Goromonzi district 2014 by (4). The difference may also be due to the fact the targeted areas in ward 13 and 14 are dominated by pole and dagga houses as compared to brick and dagga houses. It was discovered that staying in a house with open eaves had high chances of contracting malaria as compared to staying in houses without open eaves. A study in Beitbridge, Zimbabwe, showed that those that were exposed to houses with open eaves were more likely to contract malaria than those that did not (5).

Apart from staying in a house with open eaves, having frequent routine outdoor activities in the evening and at night was found to be statistically associated with contracting malaria. For instance, night fishing. Such is also in line with the malaria outbreak investigation by Tim Freeman, in Mberengwa (7). In this study, it was also learnt that most common outdoor evening and night activities were bathing and fishing in Budzi River and the swamp in Machowiro village. Although it had rained just once the water table was still far low such that most community members continued to rely on Budzi River and Machowiro swamp for laundry and domestic water uses.

Staying within the radius of 3km from a river or dam or a perennial swamp was discovered to be significantly associated with contracting Malaria. Those that stayed within that zone had odds of 2.86 of contracting malaria as compared to those that stayed more than 3km away from such water sources. The results of the association of staying close to such water sources and contracting malaria are similar to the findings in the outbreak investigation 2005 in South Andamana, India (8,9). The study reviewed that those that stayed in houses close to the Tsunami created ponds were at high risk of contracting malaria. This is because such water sources are a breeding environment for mosquitoes.

In this study, it was discovered that having no ITN hanged in the room was associated with odds of 2.71 of developing malaria compared to having an ITN hanged in the room. A malaria outbreak investigation study in Beitbridge also showed that not having an ITN hanged in the room was significantly associated with contracting Malaria (5).

The study found that of those that had visited Mozambique 6 week before the illnesses there were more cases than controls. The risk of contracting malaria was 1.945 times higher in those visited Mozambique than those that had not gone to Mozambique before the illness. Discussions with community members and nurses at Kopera Clinic brought out that some residents of ward 13 and 14 frequent Mozambique on daily basis for various reasons, which include trade, farming, firewood, and family visits. Mozambique has a high prevalence of malaria.

### Knowledge of participants on malaria

Generally, the knowledge levels of Malaria signs and symptoms were high amongst the cases and controls. Above 50% of each group could recall at least 3 signs and symptoms of Malaria. This also supports the point that the Nurses at Kopera Clinic had already conducted awareness campaigns on Malaria following the emergence of the outbreak. Having low knowledge level of Malaria signs and symptoms was found to be a risk of contracting malaria. Those that had low knowledge of malaria signs and symptoms were 2.11 times more likely to develop malaria compared to those that had high knowledge levels of the signs and symptoms of Malaria.

Such trends are similar to the findings of the study done in Muleba district, Tanzania. The study revealed that low knowledge level about malaria signs and symptoms predisposed community members to malaria (6).

Another study on knowledge practices and perceptions about malaria in rural communities of Zimbabwe also had the same perspective. The study discovered that 50% of participants had no clear understanding of the objective of the IRS programme, and this lack of knowledge had led to resisting having houses sprayed (9,15). The only difference with the two studies is that this study measured knowledge level of malaria signs and symptoms whilst the other study assessed knowledge on the objectives of the IRS programme.

### Knowledge of Malaria prevention

Most respondents (89% cases and 83% controls) were aware of the fact that sleeping under an ITN prevents one from contracting malaria. ITN prevents mosquito bites thereby reducing one’s chances of contracting malaria. However, such a high knowledge of the use of ITN as a malaria prevention strategy is contradictory to the practice of the use of ITN amongst the respondents. Out of the 46 cases and 46 controls, 70% of cases had no hanged ITN in their rooms and 21% of controls had the same scenario. One could assume that the respondents could be having high knowledge on the use of ITNs but the availability of ITNs could be very low.

Apart from knowledge on ITNs as malaria prevention, there was a significant fraction of respondents who pointed out that avoiding eating mango fruits can be a malaria prevention strategy.

### Health facility preparedness for the outbreaks

Generally, the health center was well prepared for the outbreak; this was shown by the availability of most essentials above the minimum stock. Such include the RDT check kits and malaria treatment drugs.

However, in this period of the outbreak week 30 to week 40 of 2015, it was discovered that not all the households had been covered by Indoor Residual Spraying (IRS). The houses had been last sprayed in year 2012 and it was also learnt that in the 2012 the IRS did not cover all households for various reasons; one of them was that some IRS team members were barred from reaching all households due to the too steep terrain.

## Conclusion

The outbreak investigation revealed that staying in a house with open eaves, staying within 3 km of a dam or a swamp, not having an ITN hanged in the room and having visited Mozambique 6 weeks before the illness was significantly associated with contracting malaria. Having routine outdoor activities in the evening and having evening meals outdoors was also associated with contracting malaria but the association was not statistically significant. Consequently, having a low knowledge on signs and symptoms of malaria predisposed people to contracting malaria. There is high need to intensify all pillars in the malaria prevention and control programs and maintenance of a strong surveillance system to prevent future occurrences of outbreaks.
